# Formulation of Liver-Specific PLGA-DY-635 Nanoparticles Loaded with the Protein Kinase C Inhibitor Bisindolylmaleimide I

**DOI:** 10.3390/pharmaceutics12111110

**Published:** 2020-11-18

**Authors:** Blerina Shkodra, Adrian T. Press, Antje Vollrath, Ivo Nischang, Stephanie Schubert, Stephanie Hoeppener, Dorothee Haas, Christoph Enzensperger, Marc Lehmann, Petra Babic, Kay Jovana Benecke, Anja Traeger, Michael Bauer, Ulrich S. Schubert

**Affiliations:** 1 Laboratory of Organic and Macromolecular Chemistry (IOMC), Friedrich Schiller University Jena, Humboldtstrasse 10, 07743 Jena, Germany; blerina.shkodra-pula@uni-jena.de (B.S.); antje.vollrath@uni-jena.de (A.V.); ivo.nischang@uni-jena.de (I.N.); s.hoeppener@uni-jena.de (S.H.); dorothee.haas@uni-jena.de (D.H.); anja.traeger@uni-jena.de (A.T.); 2 Jena Center for Soft Matter (JCSM), Friedrich Schiller University Jena, Philosophenweg 7, 07743 Jena, Germany; stephanie.schubert@uni-jena.de (S.S.); michael.bauer@med.uni-jena.de (M.B.); 3 Department of Anesthesiology and Intensive Care Medicine, Nanophysiology Group, Jena University Hospital, Am Klinikum 1, 07747 Jena, Germany; adrian.press@med.uni-jena.de (A.T.P.); petra.martinac@gmail.com (P.B.); jovana.benecke@googlemail.com (K.J.B.); 4 Center for Sepsis Control and Care, Jena University Hospital, 07747 Jena, Germany; 5 Institute of Pharmacy, Department of Pharmaceutical Technology and Biopharmacy, Friedrich Schiller University Jena, Lessingstrasse 8, 07743 Jena, Germany; 6 SmartDyeLivery GmbH, Botzstrasse 5, 07743 Jena, Germany; christoph.enzensperger@smartdyelivery.de (C.E.); marc.lehmann@smartdyelivery.de (M.L.)

**Keywords:** active targeting, bisindolylmaleimide I, drug delivery, liver disease, nanoparticle formulation, PKC, PLGA nanoparticles, theranostics

## Abstract

Bisindolylmaleimide I (BIM-I) is a competitive pan protein kinase C inhibitor with anti-inflammatory and anti-metastatic properties, suggested to treat inflammatory diseases and various cancer entities. However, despite its therapeutic potential, BIM-I has two major drawbacks, i.e., it has a poor water solubility, and it binds the human ether-à-go-go-related gene (hERG) ion channels, potentially causing deadly arrhythmias. In this case, a targeted delivery of BIM-I is imperative to minimize peripheral side effects. To circumvent these drawbacks BIM-I was encapsulated into nanoparticles prepared from poly(lactic-*co*-glycolic acid) (PLGA) functionalized by the near-infrared dye DY-635. DY-635 served as an active targeting moiety since it selectively binds the OATP1B1 and OATP1B3 transporters that are highly expressed in liver and cancer cells. PLGA-DY-635 (BIM-I) nanoparticles were produced by nanoprecipitation and characterized using dynamic light scattering, analytical ultracentrifugation, and cryogenic transmission electron microscopy. Particle sizes were found to be in the range of 20 to 70 nm, while a difference in sizes between the drug-loaded and unloaded particles was observed by all analytical techniques. In vitro studies demonstrated that PLGA-DY-635 (BIM-I) NPs prevent the PKC activation efficiently, proving the efficacy of the inhibitor after its encapsulation, and suggesting that BIM-I is released from the PLGA-NPs. Ultimately, our results present a feasible formulation strategy that improved the cytotoxicity profile of BIM-I and showed a high cellular uptake in the liver as demonstrated in vivo by intravital microscopy investigations.

## 1. Introduction

Bisindolylmaleimide I (BIM-I)—also known as *Gö* 6850 or GF 109203X—is a potent drug molecule with a half-maximal inhibitory concentration (IC_50_) of 10 nmol L^−1^, binding on the ATP-binding site of the α, β1, β2, γ, δ, and ε isoforms of the protein kinase C (PKC) [1,2]. PKCs are a family of serine/threonine isoenzymes that are expressed in various tissues—including the liver, heart, brain, muscle, skin, kidney, lungs, and lymphoid—thus, they are also involved in the regulation of various physiological and pathophysiological cellular functions [3,4]. The ubiquitous cellular localization of the PKCs and their participation in different signal transduction processes, explains the increased interest to investigate these kinases as suitable pharmacological targets [4]. Numerous studies have reported on treatment strategies for various diseases, implicating either the activation or inhibition of the PKC [5,6,7]. Furthermore, in chronic liver injury, PKC inhibition may prevent the transformation of hepatic stellate cells (HSCs) into unhealthy fibrotic cells, thereby preventing further propagation of the liver damage [8,9]. There is also ample evidence indicating that PKC activation is associated with impairments of bile formation and/or secretion into the hepatocytes, thus resulting in cholestasis [10,11,12,13]. Among other PKC inhibitors, BIM-I is generally known for its high selectivity toward the PKC, which has been shown to be involved in preventing inflammatory events caused by PKC activation [14]. In addition, other studies have reported that BIM-I elicits anticancer activity by various mechanisms, such as halting the progression of melanoma by inhibiting the 3-phosphoinositide-dependent protein kinase-1 (PDPK1) [15,16], and impeding multi-drug resistance by either binding to P-glycoproteins [17] or inhibiting the release of exosomes and microvesicles [18]. Another PKC inhibitor (Midostaurin) recently entered the pharmaceutical market for the adjunctive therapy of acute myeloid leukemia [19]. Therefore, considering the high mortality rates of liver disease and cancer [20,21,22], it is important to emphasize that the anti-inflammatory role of BIM-I in liver cells, combined with its anticancer activity, makes this PKC inhibitor an interesting drug, in particular if a cell-specific delivery can be realized. However, some studies have reported that BIM-I directly blocks human ether-à-go-go-related gene (hERG) potassium channels, thus impeding the cardiac action potentials, and potentially causing heart arrhythmias [23,24]. Furthermore, the hydrophobicity of the BIM-I (LogP of 3.83, topological polar surface area (TPSA) [25] of 73.9 A^2^) also decreases its potential for in vivo applications without appropriate enabling formulation strategies, such as encapsulation of the drug in nanoparticles (NPs).

Since PKCs are interesting pharmacological targets, it is of paramount importance to be able to investigate their role in a specific organ without eliciting peripheral effects. Hence, optimizing a nanoformulation of BIM-I for a liver-specific delivery would be beneficial in two aspects: (I) Minimizing the unwanted side effects of BIM-I on the hERG potassium channels, and (II) minimizing the peripheral effects arising from the PKC inhibition.

We previously described the encapsulation of BIM-I into non-functionalized poly(D, L-lactic-*co*-glycolic acid) (PLGA) NPs and established a formulation protocol, which produced particles of approx. 170 nm in hydrodynamic size with an encapsulation efficiency (EE) of about 60% [26]. It has been reported that NPs of 30 to 60 nm in size show maximal cellular uptake regardless of the composition of the NPs [27]. Nonetheless, to facilitate cell-specific PKC inhibition, BIM-I-loaded NPs functionalized with an active targeting moiety are required. Cell specificity of BIM-I-loaded NPs may be tuned by attaching the near-infrared dye DY-635, since it is a fluorescent ligand to organic anion transport pumps (OATPs) OATP1B1 and OATP1B3, which are expressed by hepatocytes and various types of cancers [28,29]. Thus, DY-635 functionalized NPs may support a cell-type-specific accumulation of BIM-I.

In this contribution, PLGA-DY-635 NPs loaded with BIM-I (PLGA-DY-635 (BIM-I)) with a hydrodynamic average size of less than 100 nm were prepared via nanoprecipitation. Additionally, a range of orthogonal analytical methods (dynamic light scattering (DLS), analytical ultracentrifugation (AUC), cryo transmission electronic microscopy (*cryo*-TEM)) were used for the physicochemical characterization of the NPs. The resulting nanoformulation of PLGA-DY-635 (BIM-I), which is primarily intended for parenteral administration, was further investigated. Subsequent in vitro studies demonstrated that (I) encapsulation of the PKC inhibitor into polymeric NPs significantly decreased its cytotoxicity, (II) the encapsulated inhibitor efficiently prevented the PKC activation, and (III) based on the in vivo evaluation, the NPs were highly taken up into the liver. Following our example, other drug molecules of interest possessing pharmacological activity in the liver can be formulated into biodegradable PLGA-DY-635 NPs to potentially increase their developability.

## 2. Materials and Methods

### 2.1. Materials

PLGA (Resomer RG 502 H, copolymer composition 50:50, 7 to 17 kDa, acid-terminated) was purchased from Evonik Industries, Germany. Poly (vinyl alcohol) (PVA) (Mowiol 4–88, partially hydrolyzed) and dimethyl sulfoxide (DMSO) (>99% UV-grade), 15-mL Amicon^®^ filters (100,000 g mol^−1^ MWCO) were purchased from Sigma-Aldrich, Steinheim, Germany (now Merck, Darmstadt, Germany). The PKC inhibitor (BIM-I) was purchased from Tocris Bioscience, Wiesbaden-Nordenstadt, Germany. DY-635-Amine was purchased from Dyomics, Jena, Germany. PLGA (Resomer RG 502 H) was covalently coupled with DY-635 via the EDC-NHS chemistry (EDC, 1-Ethyl-3-(3-dimethylamino-propyl)-carbo-diimide; NHS, *N*-hydroxysuccinimide). The dye-coupling was done as described previously with a slight modification to the procedure [28], and was kindly provided by SmartDyeLivery GmbH, Jena, Germany via a material transfer agreement. The final degree of dye functionalization was around 1 µg dye mg^−1^ polymer.

### 2.2. Nanoparticle Formulation

Nanoprecipitation was used for the NP formulation. PLGA-DY-635 (60 mg) was dissolved in 9.4 mL of DMSO. For the drug, a stock solution of 5 mg mL^−1^ BIM-I in DMSO was prepared, from which 0.6 mL were mixed with the polymer solution. The organic mixture was added at a flow rate of 2 mL min^−1^ using a syringe pump (Aladdin AL1000-220, World Precision Instruments, Berlin, Germany) into 80 mL of a 0.3% (*w/v*) PVA solution under continuous stirring at 800 rpm and at room temperature. For the preparation of larger volumes of NP formulations, the procedure described above was repeated and the yields pooled to reach the desired application volumes.

Ultrafiltration was used to purify the NPs from free drug and DMSO using a Rotina 380R Centrifuge (Hettich Lab Technology, Tuttlingen, Germany). The NP dispersion (90 mL) was transferred into six 15-mL Amicon^®^ filters (100,000 g mol^−1^ MWCO) and centrifuged on a swing-bucket at 4500× *g* for 30 min (NPs without BIM-I) or 60 min (NPs with BIM-I), until the retentate volume in the filter was 250 to 500 µL. Further, the retentate volumes from five Amicon^®^ filters were transferred into the sixth Amicon^®^ filter and filled up to 15 mL with pure water. The next centrifugation was done for 60 (NPs without BIM-I) and 80 min (NPs with BIM-I), until the retentate volume in the filter was 0.5 or 1 mL, respectively. Finally, the retentate volumes of NPs were redispersed in 6 mL of pure water, vortexed for 5 to 10 s, and stored at 4 °C overnight for complete redispersion.

Crossflow filtration or tangential flow filtration (SARTOFLOW^®^ Slice 200 Benchtop Crossflow System, Sartorius GmbH, Göttingen, Germany) was used to purify the NPs. A Hydrosart^®^ 200 filtration cassette membrane with a molar mass cut-off of 100,000 g mol^−1^ was used (Sartorius GmbH, Göttingen, Germany). Although the membrane is resistant to 10% (*v/v*) DMSO, the NP formulations were diluted 1:2 with pure water to have < 5% (*v/v*) DMSO in the dispersions. The filtration process was performed at room temperature with a flow rate of 154 mL min^−1^ for 60 to 90 min. The removal of free BIM-I from the NP dispersions was monitored by measuring the fluorescence of the filtrate (λ_ex_ = 440 nm and λ_em_ = 660 nm), i.e., 5-mL samples of the filtrate were taken every 10 min for 60 min in total. The concentration of the free BIM-I in the filtrate reached a plateau of 0.75 µg mL^−1^, and the NP formulation reached a theoretical concentration of around 25 mg mL^−1^ of the NPs. Ultimately, all NP dispersions were filtered with sterile cellulose acetate (CA) syringe filters (pore size 0.45 µm) under a sterile biosafety cabinet. The NP dispersions were stored at 4 °C.

### 2.3. Dynamic Light Scattering (DLS)

DLS was performed to determine the size, polydispersity index (PDI), and the zeta potential (ζ) of the NPs, utilizing a Zetasizer Nano ZS (Malvern Instruments, Herrenberg, Germany). NP dispersions were diluted to around 0.2 mg mL^−1^ and measured five times at 25 °C with a 30 s equilibration between the measurements. Particle sizes are expressed as the hydrodynamic diameter (d_H_) from the intensity-weighed distribution calculated using the cumulants fit method from the Malvern Zetasizer software. The volume- and number-weighted distributions are also reported, converting the intensity-weighted size by applying the Mie theory [30,31]. NPs were characterized after nanoprecipitation, after purification, and after sterile filtration.

### 2.4. Cryo Transmission Electron Microscopy (cryo-TEM)

*Cryo*-TEM investigations were conducted on a Technai G² 20 transmission electron microscope from FEI facilitated with a LaB_6_ filament. Images were acquired at an acceleration voltage of 200 kV. The investigations were conducted on Quantifoil grids (R2/2, Quantifoil, Großlöbichau, Germany). Samples were prepared in a FEI Vitrobot Mark IV (blotting time: 1 s; blotting offset −3.5 mm) utilizing 8.5 µL of the sample solution, which was deposited onto the Quantifoil grid. Samples were vitrified by plunging the grid into liquid ethane. After the transfer of the grids from the Vitrobot into the Gatan 626 cryo holder, utilizing the Gatan cryo stage, samples were transferred into the TEM, taking care that the temperature remained on a level below −175 °C. Images were acquired with a MegaView CCD camera system (Olympus Soft Imaging Solutions, OSIS, Japan) or a 4k Eagle CCD camera. Image processing was performed with ImageJ where 80 particles per image were analyzed.

### 2.5. Analytical Ultracentrifugation (AUC)

Sedimentation velocity experiments were performed using a ProteomeLab XL-I analytical ultracentrifuge (Beckman Coulter Instruments, Brea, CA, USA). The An-50 Ti eight-hole rotor, containing cells with double-sector epon centerpieces with a 12-mm optical solution path length, was spun at 5000 rpm. All experiments were performed for suitable timescales to finish NP sedimentation and radial refractive index and absorbance scans were acquired at 10 min intervals. The cells were filled with 440 μL of the diluted sample in water and with 470 μL of water as the reference. Interference and absorbance optics detection in terms of optical density (OD) at two different wavelengths, i.e., λ = 462 nm (representative of the encapsulated drug) and λ = 635 nm (representative of the targeting dye), was used for observation of the sedimentation boundary in respect to time. A suitable selection of scans was used for data evaluation with least squares sedimentation boundary modelling. Therefore, the ls-g*(s) model from Sedfit without considering effects of diffusion was utilized [32]. To calculate hydrodynamic sizes, the relation $d\left(h,\;NP\right)=3\sqrt{2}\sqrt{\left[s\right]v_{NP}}$ was used by assuming a partial specific volume of the NPs of $\upsilon \left(NP\right)=0.77\;{\mathrm{cm}}^{3}g^{-1}$ and a determined intrinsic sedimentation coefficient [s], calculated from the present data [33].

### 2.6. UV–VIS Spectroscopy

UV–VIS spectroscopy was carried out using the Infinite M200 Pro Platereader (Tecan Group, Männedorf, Switzerland) for the investigation of the encapsulation efficiency of BIM-I in the NPs. From each formulation, aliquots were taken after the sterile filtration and lyophilized. The lyophilized NPs were dissolved in DMSO and absorbance was measured at λ = 462 nm. Standard curves of BIM-I were obtained using the same measurement protocol. All measurements were done on a 96-well flat-transparent quartz plate (Hellma, Jena, Germany). The loading capacity (LC) and the EE were calculated with the following formulas:

LC = (mass of drug recovered)/(mass of total particle recovered − mass of surfactant) × 100,

EE = (LC found)/(LC theoretical) × 100.

UV–VIS spectroscopy was also used to quantify the residual amount of PVA (%, *w/w*) in the NP dispersions. The assay is based on the complex formation of PVA with iodine/potassium iodide (i.e., Lugol’s solution) [34]. In total, 90 µL of NP dispersion were pipetted on a 96 well-plate and the following reagents are added in sequence: 10 µL of 1 M sodium hydroxide to degrade the particles (15 min shaking at 850 rpm, at room temperature); 10 µL of 1 M hydrochloric acid to neutralize the resultant solutions; 60 µL of 0.65 M boric acid; and 10 µL Lugol’s solution. The measurements were done at λ = 650 nm after 15 min of addition of the Lugol’s solution. A calibration curve was obtained and used to determine the concentration of the drug in the NP samples.

### 2.7. High-Performance Liquid Chromatography (HPLC)

An Agilent Technologies 1200 Series instrument was used with a C18 column Zorbax Eclipse XDB-C18, 4.6 × 150 mm, 5 µm (Agilent Technologies, Waldbronn, Germany). A stock solution of 1 mg mL^−1^ BIM in DMSO was prepared with further serial dilutions. Standard solutions (50 µL) were further dissolved in 950 µL of an acetonitrile/0.1% trifluoroacetic acid (TFA) mixture. The solutions were filtered with 0.45-µm PTFE syringe filters into HPLC vials, and 10 µL of sample solution were injected. For the preparation of NP solutions, at least 1 mg of lyophilized NPs was dissolved in DMSO and sonicated until being completely dissolved. Analytically, the dissolved NP solutions were treated the same way as the standards. For the HPLC measurements, a gradient of 80% eluent A (water 0.1% TFA) and 20% eluent B (acetonitrile 0.1% TFA) was used. Detection of BIM-I was done via the absorbance detector at λ = 448 nm.

### 2.8. Cell Viability Assay

Cell viability studies were performed with the mouse fibroblast cell line L929 (CLS, Eppelheim, Germany), as recommended by ISO10993-5. Further, the murine hepatoma cell line Hepa1-6 (DSMZ no.: ACC 175) as well as the human liver carcinoma cell line HepG2 (CLS, Germany) were used. The L929 and Hepa1-6 cells were cultured in Dulbecco’s modified eagle’s medium (DMEM) supplemented with 1 or 4.5 g L^−1^ glucose, respectively. The HepG2 cells were cultured in Ham’s F12 medium (Biochrome), supplemented with 10% (*v/v*) fetal calf serum (FCS, Capricorn), 100 U mL^−1^ penicillin, and 100 µg mL^−1^ streptomycin at 37 °C in a humidified 5% (*v/v*) CO_2_ atmosphere. In detail, cells were seeded at 10^4^ cells per well in a 96-well plate without using the outer wells. Then, 1 h after medium exchange, cells were treated in sextuplicates with NPs and drug at indicated concentrations and were incubated for an additional 24 h. The medium was replaced by a 10% (*v/v*) AlamarBlue solution in fresh culture medium, prepared according to the manufacturer’s instructions. Following an incubation for 4 h at 37 °C, the fluorescence intensity was measured at λ_Ex_ = 570/λ_Em_ = 610 nm. Non-treated cells on the same plate were referred to as 100% viability. Values below 70% were regarded as cytotoxic. Data are expressed as mean ± SD of at least three independent determinations.

### 2.9. Animals and Intravital Microscopy

FVB/N mice (male and female, minimum 8 weeks old) are bred and housed in the animal facility of the Jena University Hospital under specific pathogen-free conditions. Intravital microscopy was performed on an LSM-780 in a semi-sterile environment as described previously [28] with some adaptation. In brief, differently to the previously described method and beside the general anesthesia induced using Isoflurane, animals received an additional Metamizol analgesia (2.5 mg per animal, oral). All animal protocols were approved in advance by the Thuringian state administrative office and the state-appointed ethical committee (Project No. 02-026/16).

### 2.10. Phospho-PKC Substrate Western Blot

HepG2 cells were cultivated in DMEM/F12, 10% (*v/v*) FCS, and 1% (*v/v*) penicillin/streptomycin in a humidified atmosphere (37 °C, 5% CO_2_). Stimulations were carried out in serum-free DMEM/F12. Cells seeded for 24 h on 12-well plates were starved for approximately 3 h in serum-free DMEM/F12. Then, 30 min before stimulation, DMSO, 200 nmol L^−1^ BIM-I, or NPs (equal to approximately 200 nmol L^−1^ BIM-I) were added. Cells were stimulated with phorbol 12-myristate 13-acetate (PMA, Tocris/ Biotechne, Wiesbaden, Germany) for another 15 min, washed twice with phosphate-buffered saline (PBS) (without calcium and magnesium), and immediately lysed in radioimmunoprecipitation (RIPA) buffer containing protease and phosphatase inhibitors (Phosphostop 2, Roche, Mannheim, Germany and Halt Protease inhibitor cocktail, Thermo Fischer Scientific, Dreireich, Germany). Protein concentrations were determined by bicinchoninic acid assay (BCA Macro Kit, Serva Electrophoresis GmbH, Heidelberg, Germany). For the detection of the PKC activity, approximately 8 µg of each sample were diluted with 2 × Laemmli sample buffer (Serva Gelelectrophoresis GmbH) and loaded on pre-casted HSE gels (Serva Electrophoresis GmbH, Heidelberg, Germany). Proteins were separated at 120 V for approximately 1.5 h in Laemmli Running Buffer (Serva Electrophoresis GmbH, Heidelberg, Germany) and transferred on a polyvinylidene difluoride (PVDF) membrane using semi-dry blotting (PowerBlot, ThermoFischer Scientific, Dreireich, Germany). Afterwards, gels were stained in PageBlue protein staining solution (Thermo Fischer Scientific, Dreireich, Germany) and imaged on a gel documentation system (GeneSnap, Syngene, Dreireich, Germany). PVDF membranes were incubated in 5% bovine serum albumin (BSA) in TBS-Tween for 1 h, followed by 1 h of phospho-PKC (pPKC) substrate antibody (Cell Signaling Technologies, 6967S, RRID:AB_10949977 diluted 1:1000 in 5% BSA in TBS-Tween). The membrane was then washed in TBS-Tween followed by incubation for 1 h in horseradish peroxidase (HRP) conjugated anti-rabbit antibody (Jackson Immuno Research, 711-036-152, RRID:AB_2340590, 1:5 000 in 5% BSA, TBS-Tween). After two more times of washing in TBS-Tween, Luminol (SERVALight Eos CL HRP WB Substrate, Serva Gelelectrophoresis GmbH, Heidelberg, Germany) was added and the signals collected on a LAS 3000 ImageQuant. The contrast was adjusted for better visualization using the FIJI software [35]. Image processing as stated was applied to the whole image, if otherwise not stated, to maintain contrast differences within the blot. Densitometric analysis of both, Coomassie-stained gels and the Western blots (whole lanes), was performed using ImageJ (Gel Analyzer Plugin, ImageJ v1.51 [36]). The signal from the pPKC substrate Western blot was normalized to the gel loading (Coomassie staining). Data are expressed relative to the control (15 min DMSO without inhibitors or nanocarrier) for each individual Western blot and averaged afterwards.

## 3. Results and Discussion

For the formulation of BIM-I-loaded NPs, PLGA was chosen as the polymeric carrier material due to its beneficial properties such as excellent biocompatibility and biodegradability [37]. PLGA with uncapped carboxylic acid chain termini was used to formulate the NPs for two reasons:

(I) The reliable attachment of DY-635 to the carboxylic acid chain termini of the polymer for enhanced uptake into hepatocytes, and (II) because of the ionic attraction between the potential positively charged dimethylaminopropyl sidechain of BIM-I with the potential negatively charged carboxylic acid chain termini, which could increase the encapsulation efficiency [38]. Therefore, to identify a formulation method and conditions that result in NPs of ≤ 100 nm in size for an optimized targeted uptake, different formulation techniques were screened in preliminary experiments (Supplementary Materials, Figure S1A). Nanoprecipitation was found to be the most suitable method to obtain small NPs with monomodal size distributions since other techniques, such as nanoemulsion or microfluidic approaches, result in larger particle sizes (>200 nm) and PDIs (Supplementary Materials, Figure S1A) [39,40]. Moreover, based on our data and the reported literature, the choice of the organic solvent in NP preparation also influences the size of the NPs significantly (Supplementary Materials, Figure S1B) [41]. The particle formation during the nanoprecipitation method is governed by a spontaneous diffusion-driven mechanism that is highly influenced by the mixing dynamics of the polymer between the organic solvent and the aqueous phase [42]. In this regard, the solvent-water miscibility affects the size of the formed particles, i.e., organic solvents possessing a higher miscibility with water (e.g., DMSO > acetone) facilitate a more efficient mass transfer of the polymer to the aqueous phase, triggering a fast nucleation of the polymer solutes and thus promoting the formation of smaller NPs [41,42].

**Figure 1:**
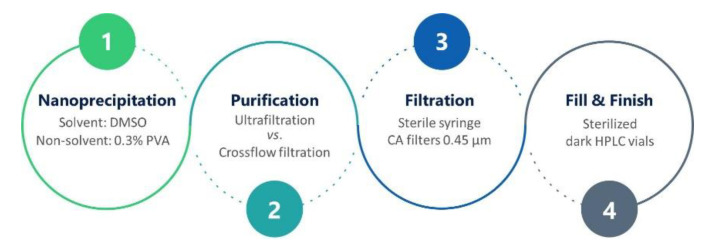
Schematic representation of the formulation process of NPs. *CA* denotes cellulose acetate.

Thus, in contrast to our previous formulation protocol, where acetone was used as the organic solvent [26], here, DMSO was used since it yields smaller NPs [43]. Ultrafiltration and crossflow filtration were utilized to remove DMSO and free BIM-I from the NP formulations, since the sedimentation of NPs via centrifugation was not possible due to the DMSO providing a higher density than the NPs in water. Crossflow filtration was employed for upscaling purposes to prevent the formation of a filter cake [44], and the results were compared to the standard ultrafiltration method. For all formulations, PVA with a nominal molar mass of 31,000 g mol^−1^ was used to provide a stable dispersion and maintain the NP integrity during the formulation procedure. An optimized formulation and purification protocol for BIM-I-loaded PLGA-DY-635 NPs with a diameter below 100 nm was developed. A step-by-step overview of the formulation and downstream process is shown in Figure 1.

### 3.1. Nanoparticle Characterization

#### Size, Population Distribution, and Surface Charge

**Figure 2:**
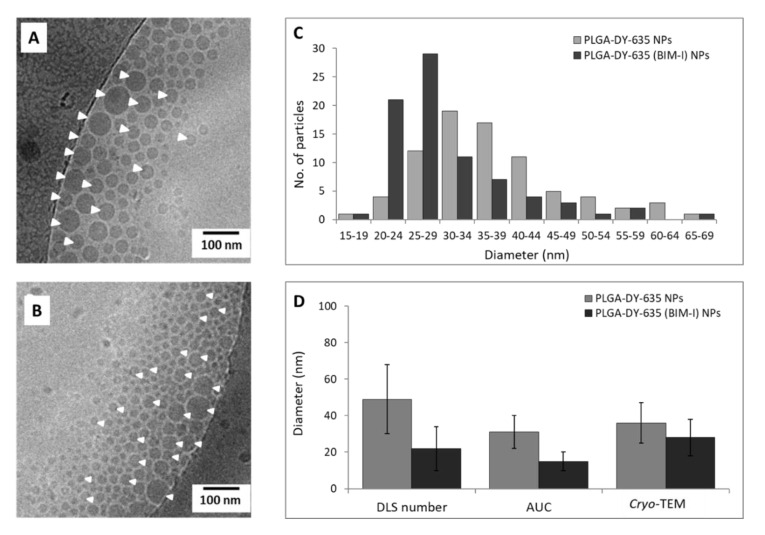
*Cryo*-TEM images of PLGA-DY-635 NPs (**A**) and PLGA-DY-635 (BIM-I) NPs (**B**) purified via crossflow filtration (white arrow heads point to NPs); particle analysis from *cryo*-TEM images was carried out by ImageJ software and histograms represent the number distribution (*n* = 80) (**C**); Diameter of the NPs purified via the crossflow filtration estimated by the different analytical methods (**D**). The size variation bars represent the standard deviation of sizes calculated from the respective distributions of sizes, i.e., of the number-based hydrodynamic diameter distributions from a representative distribution of the five DLS measurements, the hydrodynamic diameter distributions derived from differential distributions of sedimentation coefficients, and the mean apparent geometrical NP diameter from *cryo*-TEM measured by ImageJ.

PLGA-DY-635 (BIM-I) NPs were prepared batch-wise and were characterized regarding their apparent NP sizes, PDIs, zeta potential by DLS (Table 1), the morphology and size obtained from *cryo*-TEM imaging (Figure 2), as well as the drug and surfactant concentration obtained from UV-VIS spectroscopy (Table 2). For the ultrafiltrated NPs, the intensity distributions from DLS revealed that the unloaded NPs feature an apparent hydrodynamic diameter of 120 nm and the drug-loaded NPs were around 92 nm in apparent hydrodynamic diameter (Table 1). Meanwhile, particles purified via crossflow were 65 and 63 nm for the unloaded and the drug-loaded NPs in apparent hydrodynamic diameter, respectively. Occasionally, we observed noisy intensity correlation functions, presumably caused by the encapsulated drug and targeting dye molecules. Both are optically active with excitation wavelengths of 462 and 635 nm, respectively. The latter is near the 633-nm laser wavelength of the DLS to record intensity fluctuations utilized for the calculation of hydrodynamic sizes. In this case, the NPs’ absorbance and fluorescence may interfere with the laser wavelength [45,46]. This was observed by comparing the correlograms of PLGA NPs and concentrated vs. diluted PLGA-DY-635 NPs, which showed that samples without and/or a lower dye concentration showed fewer signal anomalies (Supplementary Materials, Figure S2). Furthermore, considering the fact that differently sized particles scatter light with different intensities, where larger particles typically dominate the results [47,48], and taking into account the relatively high PDI of PLGA-DY-635 (BIM-I) NPs, the results from the volume- and number-weighted size distributions are also reported, which are more relevant in this case (Table 1). Compared to the intensity-weighted distributions, for all NPs, the volume- and number-weighted distributions showed a shift to considerably smaller sizes, which can be considered typical for disperse particle populations [49]. Nevertheless, all DLS distributions of ultrafiltrated NPs showed that the drug-loaded NPs were apparently smaller on average than the unloaded NPs by a factor of approximately two. The differences were barely observed for crossflow filtrated particles.

Considering the substantial differences between the intensity-, volume-, and number-weighted distributions from the DLS, other analytical methods, i.e., AUC and *cryo*-TEM, were employed for resolving the NP sizes (Figure 2D). AUC analysis revealed that PLGA-DY-635 and PLGA-DY635 (BIM-I) NPs purified via crossflow were 31 ± 9 and 15 ± 5 nm in apparent hydrodynamic diameter, respectively, whereas *cryo*-TEM revealed that the same NPs were slightly larger (36 ± 11 and 28 ± 10 nm, respectively). It can be seen that the results from the AUC, *cryo*-TEM, and the number distribution from DLS are in good agreement; most importantly, the BIM-I-loaded NPs were smaller on average than the unloaded NPs (Figure 2D). This size difference was found for the particle size analysis on *cryo*-TEM images and AUC investigations as well (Figure 2). Although the particle size is governed by a combination of formulation parameters (i.e., solvent interactions, stirring speed, polymer and surfactant concentration) [50,51], here, additional influences can be attributed to high polymer–dye–drug interactions, more specifically, the reduction in particle size could be a result of the attraction of the positively charged dimethylaminopropyl side chain of BIM-I with the negatively charged sulfonate group of DY-635 [52].

**Figure 3:**
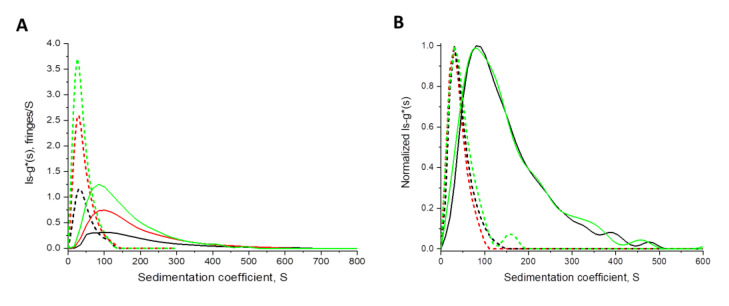
(**A**) Differential distributions of sedimentation coefficients, ls-g*(s), at three different concentrations (0.5, 1, and 1.5 mg mL^−1^ in black, red, and green, respectively) of NPs purified via crossflow filtration, (PLGA-DY-635 (BIM-I) NPs (dotted lines), PLGA-DY-635 NPs (solid lines)) using the refractive index detector. (**B**) Normalized differential distributions of sedimentation coefficients, ls-g*(s), of PLGA-DY-635 (BIM-I) NPs (dotted lines) recorded with the refractive index detector (black, polymer), absorbance detection in terms of OD at λ = 635 nm (green, targeting dye) and λ = 462 nm (red, encapsulated drug), and PLGA-DY-635 NPs (solid lines) derived utilizing the refractive index detector (black, polymer), and absorbance detection in terms of OD at λ = 635 nm (green, targeting dye). The PLGA-DY-635 NPs contain no drug (concentration of NPs was 1.5 mg mL^−1^).

In order investigate the observed size differences between the NPs and their drug-loaded counterparts in detail, we performed sedimentation velocity experiments by AUC making use of the refractive index and absorbance detection. Figure 3 shows differential distributions of sedimentation coefficients, ls-g*(s), from NPs containing dye only (solid lines) and NPs containing the drug and dye (dotted lines) at three different concentrations. By utilizing the absolute method of sedimentation measurements, it is apparent that the NPs indeed have different sizes, reflected by the substantially different distributions of sedimentation coefficients. NPs without the drug sediment at a considerably higher rate on average than NPs with the drug. This aspect of the size difference between the unloaded and loaded NPs was also indicated by the other NP sizing techniques (Figure 2D). Moreover, it could be confirmed by multi-detection in AUC that both the drug and the targeting dye are clearly contained in the NPs. This is reflected by the virtual coincidence of the normalized differential distribution of sedimentation coefficients derived from absorbance detection in terms of OD at 635 nm (DY-635, green dotted line) and at 462 nm (BIM-I, red dotted line), congruent to the refractive index detection (PLGA, black dotted line) (Figure 3B). In addition to the AUC analysis, which demonstrates that the drug was majorly incorporated in the NPs with only ca. 4% to 5% of free drug [33], the slight decrease in the zeta potential of the BIM-I-loaded NPs might also provide additional evidence for the drug incorporation in the NP matrices [53].

### 3.2. Influence of the Purification Method on the Encapsulation Efficiency

According to measurements performed by UV-VIS spectroscopy, NP formulations purified via ultrafiltration resulted in particles with a BIM-I concentration of around 200 µg mL^−1^, whereas NP formulations purified via the crossflow filtration resulted in particles with a BIM-I concentration of 419 µg mL^−1^. The drug concentration results were also confirmed by liquid chromatographic measurements (Table 2).

The difference in the drug concentration is a result of the purification method, although in both cases, the membranes were composed of cellulose-based material and had the same molar mass cut-off limit. Considering the high solubility of BIM-I in DMSO, and potentially its tendency to leave the NPs during the washing process, more BIM-I molecules may have been washed out during ultrafiltration as a result of the harsh cleaning conditions in dead-end filtrations, and due to the longer durations compared to crossflow filtration. Furthermore, the PVA content in the ultrafiltrated NPs was 20% to 30% higher than in crossflow filtrated NPs. This is due to a filter cake formation during ultrafiltration, which may clog parts of the membrane surface and decrease the overall purification efficiency [54], whereas in crossflow filtration, the tangential flow of the eluent limits the build-up of the NPs on the filter. Thus, crossflow filtration allows milder cleaning conditions that prevent drug leaking from the NPs, but at the same time is efficient enough to wash out more PVA compared to ultrafiltration. Hence, the two-fold difference of the BIM-I concentration in the NPs purified by the two different methods was also reflected in the EE, which was 32% for the ultrafiltrated NPs and nearly doubled (60%) for the crossflow filtrated NPs (Table 2). Furthermore, a two-fold difference was also observed between the diameter of the ultrafiltrated vs. crossflow filtrated PLGA-DY-635 NPs (Table 2). This could be a result of the higher amount of PVA retained in the ultrafiltrated NPs, which typically adsorbs on the surface of the NPs [55].

### 3.3. Effect of the Particles on Cell Viability

**Figure 4:**
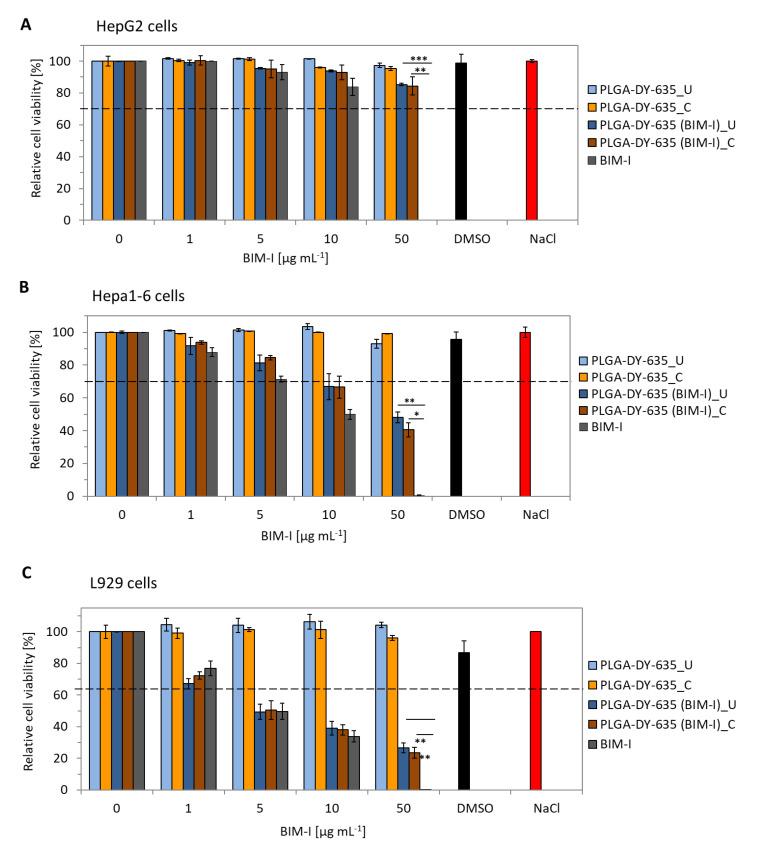
Cell viability of the NPs tested in HepG2 cells (**A**), Hepa1-6 cells, (**B**) and L929 cells (**C**); ***U*** denotes NPs purified via ultrafiltration; ***C*** denotes NPs purified via crossflow filtration. For statistical analysis, a two-tailed *t*-test was used, where *p* < 0.02 (*); *p* < 0.01 (**); *p* < 0.001 (***); *n* = 3.

Cytotoxicity investigations were conducted in three different cell lines, including hepatocytes (HepG2 and Hepa1-6), and a fibroblast cell line (L929) serving as the control. The unloaded NPs showed no cytotoxicity in the tested cell lines. The concentration of crossflow-filtered and ultrafiltered NPs was about 3 and 5 mg mL^−1^, respectively, to match the particles at BIM-I concentrations of 50 µg mL^−1^. In contrast, PLGA-DY-635 (BIM-I) NPs showed a toxic effect in Hepa1-6 and L929 cells (Figure 4) but no toxicity in HepG2 cells up to 50 µg mL^−1^ of encapsulated BIM-I. Nevertheless, compared to the 50 µg mL^−1^ free BIM-I (control), PLGA-DY-635 (BIM-I) NPs significantly improved the cell viability in all three cell lines due to slower cell internalization. In general, the NPs reduced the toxicity of the free drug by approximately 80% in HepG2 cells, 40% in Hepa1-6 cells, and 30% in L292 cells. For concentrations of less than 10 µg mL^−1^, no significant difference was observed between the free BIM-I vs. the PLGA-DY-635 (BIM-I) NPs in all three tested cell lines.

The higher cell viability observed in HepG2 cells could be due to the fact that the cells were cultured in Ham’s F12, which is a highly nutritious medium, and supplemented with serum rich in growth-promoting nutrients and proteins [56]. Thus, in contrast to the L929 and Hepa1-6 cells, which were cultured in the serum-free DMEM media [56], the supplemental proteins in HepG2 cells could have reduced the particle uptake by adsorbing to the NP surface, thus showing a higher cell viability at the measured incubation times [57,58]. On the other side, the L929 fibroblasts are non-cancerous cells with a limited proliferation that undergo senescence [59], thus showing an augmented toxicity when compared to the hepatocellular carcinoma cell lines. In general, the major differences in cell viability observed between the three cell lines can be attributed to different cell-uptake mechanisms and intracellular trafficking pathways of NPs [58] that are known to originate from several factors, including physicochemical (NP size, surface charge) and biological (protein corona, cell type, cell shape, culture medium conditions) phenomena of the cell–NP interaction [60,61].

However, although free BIM-I and PLGA-DY-635 (BIM-I) NPs seem to decrease the cell viability in vitro, it has been demonstrated that PLGA (BIM-I) NPs with a BIM-I concentration of about 85 µg mL^−1^ exhibited no toxicity when tested in an ex vivo model [26]. Furthermore, a concentration of 1.5% PVA in the NP dispersion corresponds to 15 mg mL^−1^ PVA, which, according to Menon et al., can be considered as cytocompatible when tested in breast and prostate cancer cells [62]. We have previously shown that solutions of 5% (*w/v*) PVA with a molar mass of 31,000 g mol^−1^ elicited no hemolytic activity and no erythrocyte aggregation [26].

### 3.4. Uptake of NPs in Hepatocytes In Vivo

**Figure 5:**
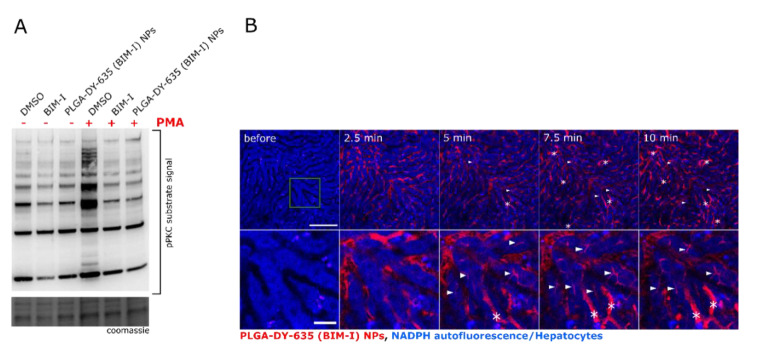
Protein kinase C inhibition by hepatocyte-directed PLGA-DY-635 (BIM-I) NPs. (**A**) DY-635 HepG2 pretreated with DMSO, free or encapsulated or BIM-I (200 nmol L^−1^) for 30 min and stimulated with PMA for 15 min. pPKC substrates were detected by Western blotting from total protein lysates and loading was evaluated by Coomassie staining of the gel. (**B**) Representative images from intravital microscopy of PLGA-DY-635 (BIM-I) NPs in the liver. Hepatocytes are detected through their strong NADPH auto-fluorescence and liver sinusoids can be identified by their negative staining (black). Lower panel depicts a zoom area (green square) of the upper panel. Some DY-635 accumulation in Kupffer cells (asterisks) was observed. DY-635-stained bile canaliculi (arrowheads) indicated the uptake and degradation of the NPs as well as elimination of DY-635. Scale bar: 0.1 mm (upper panel) and 0.025 mm (lower panel).

To test whether the PLGA-DY-635 (BIM-I) NPs are able to release their payload into the cytosol of cells and to confirm that BIM-I is actively transported via NPs, HepG2 cells were pre-incubated for 30 min with BIM-I in DMSO or with PLGA-DY-635 (BIM-I) NPs and stimulated with the strong PKC activator PMA for 15 min. The pPKC substrate antibody binds to the phosphorylated consensus amino acid sequence (RRXS*/T*) from many different proteins recognized and phosphorylated by all PKC isoforms. The resulting Western blot identifies the global amount of phosphorylated consensus sequences and thus reflects the activity of PKC isoforms [63,64] (Supplementary Materials, Table S1). Figure 5A shows that BIM-I in DMSO and PLGA-DY-635 (BIM-I) NPs prevented a PMA-mediated PKC activation efficiently in vitro, proving the efficacy of the inhibitor after its encapsulation, suggesting that BIM-I is released from the PLGA-NPs.

Moreover, NPs were investigated in vivo with intravital microscopy. This technique is beneficial for the investigation of the NP uptake since hepatocytes can be identified easily by their strong NADPH endogenous auto-fluorescence, while sinusoids—the hepatic capillaries—are non-fluorescent. Non-parenchymal cells of the liver (e.g., Kupffer cells, the local tissue macrophages) in contrast can be identified by their elongated shape and their micro-anatomical location in sinusoids. Injection of PLGA-DY-635 (BIM-I) NPs showed rapid uptake and processing of the particles in the hepatocytes, and the DY-635 label was further eliminated in the bile ducts (Figure 5B). However, non-parenchymal cells (e.g., macrophages, asterisks) also absorb NPs to a small extent. One possible reason is that a small proportion of the particles aggregated, forming larger clusters that tend to be taken up by macrophages. This shows how important the adjustment of active and passive targeting is for the efficient administration of drugs. Interestingly, during the experiments, circulating immunocompetent cells (e.g., monocytes) were not detected. The encapsulation of BIM-I in NPs with DY-635 enhanced the compartmentalization to hepatocytes (Figure 5B) compared to previously published Nile red-loaded PLGA NPs that do not carry the DY-635 targeting moiety, and which exclusively accumulated in the cells of the reticuloendothelial system (Kupffer cells) [28].

## 4. Conclusions

BIM-I exhibits anti-inflammatory properties by inhibiting multiple PKC isoforms. Numerous studies have indicated that the inhibition of the PKC has anti-cholestatic effects in hepatocytes and reduces liver inflammation, thus minimizing the severity of experimental liver fibrosis in mice. To enhance the clinical translation, we encapsulated BIM-I into PLGA NPs, which have been shown to be promising delivery carriers in improving the bioavailability of drugs with physicochemical drawbacks (i.e., poor solubility, toxicity). In addition, the incorporation of DY-635 increased the compartmentalization of the NPs in the liver, thus hinting to an active cell-type-specific targeting. Although our results demonstrate a feasible formulation strategy that improves the cytotoxicity profile of BIM-I and shows a high cellular uptake in the liver, investigations of the biodistribution of the drug, and the evaluation of the immunological side effects need to be considered before further promising applications of the NPs can be addressed. In conclusion, our NP formulation strategy may be used as an example to produce multifunctional PLGA-based delivery systems that improve the bioavailability of poorly soluble drugs and provides an enhanced cell-type-specific uptake. Additionally, utilizing biodegradable and cell-type-specific inhibitor-loaded NPs could serve as a useful tool in studies that investigate the role of the PKC in signal transduction pathways in specific organs.

## Figures and Tables

**Table 1:** Size, PDI, and zeta potential of the NPs as measured by DLS.

NP Formulation	Purification Method	d_H_ (nm) ^a^ ± SD	d_H_ (nm) ^b^ ± SD	d_H_ (nm) ^c^ ± SD	PDI ± SD	ζ (mV) ± SD
PLGA-DY-635_U	Ultrafiltration	120 ± 24	133 ± 72	66 ± 19	0.15 ± 0.07	−20 ± 8
PLGA-DY-635_C	Crossflow filtration	65 ± 17	50 ± 26	49 ± 3	0.15 ± 0.03	−20 ± 0
PLGA-DY-635 (BIM-I)_U	Ultrafiltration	92 ± 3	73 ± 2	28 ± 8	0.22 ± 0.05	−7 ± 10
PLGA-DY-635 (BIM-I)_C	Crossflow filtration	63 ± 7	37 ± 3	22 ± 2	0.30 ± 0.08	−9 ± 2

a–Z-average intensity distribution; b–volume distribution; c–number distribution. ***U*** denotes NPs purified via ultrafiltration, ***C*** denotes NPs purified via crossflow filtration. The standard deviation (SD) of samples purified via ultrafiltration represents the error between different formulated batches (*n* = 8). The standard deviation of d_H_, PDI, and ζ of the samples purified via crossflow represents the error between five DLS measurements.

**Table 2:** Concentration of the drug and PVA in the NP dispersions, as well as the EE and LC.

NP Formulation	BIM-I ^a^ (µg mL^−1^) ± SD	BIM-I ^b^ (µg mL^−1^) ± SD	EE (%) ± SD	LC (%) ± SD	NP + PVA (mg mL^−1^) ± SD	PVA (%, *w*/*v*) ± SD
PLGA-DY-635_U	/	/	/	/	18 ± 1	1.1 ± 0.1
PLGA-DY-635_C	/	/	/	/	23	0.9
PLGA-DY-635 (BIM-I)_U	192 ± 15	194 ± 42	32 ± 18	1.5 ± 1	25 ± 1	1.5 ± 0.2
PLGA-DY-635 (BIM-I)_C	419	419	60	3	24	1.0

a–determined by UV-VIS spectroscopy (*n* = 8); b–determined by HPLC (*n* = 3); PVA assay (*n* = 4). The standard deviation (SD) of samples purified via ultrafiltration represents the error between different formulated batches. ***U*** denotes NPs purified via ultrafiltration; ***C*** denotes NPs purified via crossflow filtration.

## Supplementary Materials

The following are available online at https://www.mdpi.com/1999-4923/12/11/1110/s1, Figure S1: Influence of the formulation technique and organic solvent on PLGA-DY-635 NPs; Figure S2: DLS correlograms of PLGA NPs, diluted PLGA-DY-635 NPs and concentrated PLGA-DY-635 NPs. Table S1: Densitometric analysis of the Western blots.
