# Supplementary material for: Formulation of Liver-Specific PLGA-DY-635 Nanoparticles Loaded with the Protein Kinase C Inhibitor Bisindolylmaleimide I
Source: Pharmaceutics. 2020 Nov 18;12(11):1110. doi: 10.3390/pharmaceutics12111110 (PMC7698893; doi:10.3390/pharmaceutics12111110)
Supplement: Supplementary file 1 [file pharmaceutics-12-01110-s001.pdf]

pharmaceutics

Supplementary Materials: Formulation of Liver-Specific PLGA-DY-635 Nanoparticles Loaded with the Protein Kinase C Inhibitor Bisindolylmaleimide I

Blerina Shkodra, Adrian T. Press, Antje Vollrath, Ivo Nischang, Stephanie Schubert, Stephanie Hoeppener, Dorothee Haas, Christoph Enzensperger, Marc Lehmann, Petra Babic, Kay Jovana Benecke, Anja Traeger, Michael Bauer and Ulrich S. Schubert

1. Formulation Methods

1.1. Nanoemulsion

Nanoparticles (NPs) were prepared by the single emulsion oil-in-water (o/w) method using a high power ultrasound sonicator (200 W for 20 s). A solution consisting of 5 mg mL^{-1} polymer (2 mL) in ethyl acetate was mixed with 3% (w/v) PVA solution (0.5 mL). The solution was sonicated and the resulting nanoemulsion was transferred into 20 mL pure water and then stirred for 24 h to evaporate the organic solvent.

1.2. Microfluidics

Formulation of the NPs *via* microfluidics was achieved using a neMESYS syringe pump from Cetoni, Germany. The organic phase consisting of 5 mg mL^{-1} of PLGA-DY-635 polymer in acetone, and a 0.03% (w/v) aqueous solution of PVA, were filled into the pump syringes. The pump was connected to an active micromixer chip purchased from Microfluidic ChipShop, Germany. NPs were formulated in the 80 µL volume of the chip chamber under continuous stirring at a flow rate of 3000:300 µL/min of the aqueous:organic phase.

1.3. Crossflow

The crossflow device is a prototype machine for the formulation of NPs designed by Analytik Jena, Germany. The device consists of syringe pumps connected *via* tubing, which circulate and mix liquids through a crossflow junction. PLGA-DY-635 NPs were formulated by mixing a 2.5 mL of 5 mg mL^{-1} polymer solution with 10 mL of 0.03% (w/v) solution of PVA through the crossflow junction, with a flow rate of 325 µL/min for the polymer solution, and 1.5 mL for the PVA solution.

2. Additional results

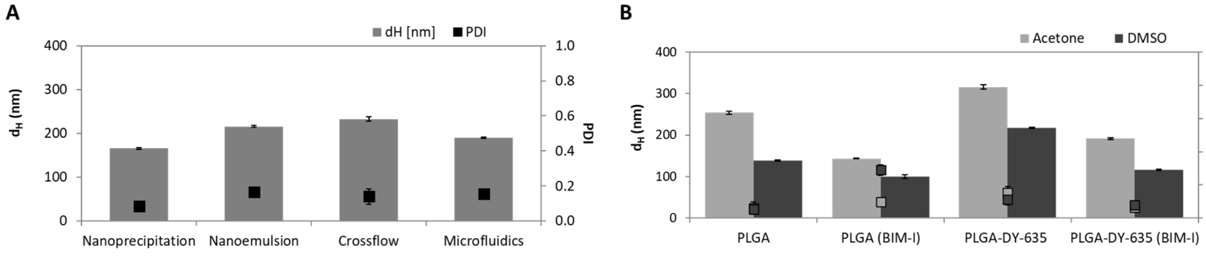

Figure S1. DLS measurements of particle size and PDI show the influence of the formulation method on PLGA-DY-635 NPs (A) and the influence of organic solvent (B) on PLGA and PLGA-DY-635 NPs formulated via nanoprecipitation. Error bars represent the standard deviations from five measurements.

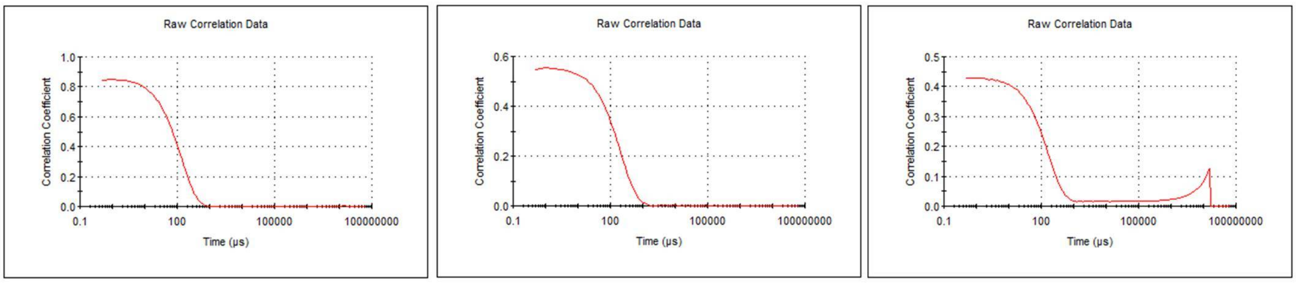

Figure S2. DLS correlograms of PLGA NPs (left), diluted (to 0.2 mg mL^{-1}) PLGA-DY-635 NPs (middle) and concentrated PLGA-DY-635 NPs (right).

2.1. Densitometric Analysis of the Western Blot

Densitometric analysis of both, coomassie stained gels and the Western blots (whole lanes) were performed using ImageJ (Gel Analyzer Plugin, ImageJ v1.51). The signal from the pPKC substrate Western blot was normalized to the gel loading (coomassie staining). Data are expressed relatively to the control (15 min DMSO without inhibitors or nanocarrier) for each individual Western blot and averaged afterwards.

Table S1. Densitometric analysis of the Western blots.

Inhibitor	Stimulation	MW	SD	N
DMSO	15' DMSO	1.00	0.00	4
BIM-I	15' DMSO	0.76	0.21	4
PLGA-DY635 (BIM-I)	15' DMSO	0.87	0.12	3
DMSO	15' PMA	1.72	0.37	5
BIM-I	15' PMA	0.87	0.18	4
PLGA-DY635 (BIM-I)	15' PMA	0.78	0.26	4

MW – Mean, SD – Standard deviation, N – Repetitions (individual stimulated wells).
